# Feeding broiler chicks with *Schizosaccharomyces pombe*-expressed phytase-containing diet improves growth performance, phosphorus digestibility, toe ash, and footpad lesions

**DOI:** 10.5713/ab.21.0462

**Published:** 2022-04-30

**Authors:** De Xin Dang, Seong Guk Chun, In Ho Kim

**Affiliations:** 1 Department of Animal Resource and Science, Dankook University, Cheonan 31116, Korea

**Keywords:** Broiler Chick, Footpad Lesion, Phosphorus Digestibility, Phytase, Toe Ash

## Abstract

**Objective:**

The objective of this study was to evaluate the effects of dietary supplementation of *Schizosaccharomyces pombe* (*S. pombe*) -expressed phytase on growth performance, apparent ileal digestibility, organ indexes, meat quality, toe ash, and footpad lesions score in broiler chicks.

**Methods:**

A total of 390 one-day-old broiler chicks were randomly assigned to 5 groups based on the initial body weight (42.15±0.17 g), there were 6 replicate cages per treatment and 13 birds (mixed sex) per cage. The experimental period was 45 days, including 4 periods (starter, days 1 to 10; grower, days 11 to 24; finisher 1, days 25 to 38; finisher 2, days 39 to 45). Dietary treatments were based on a corn-soybean meal-basal diet and supplemented with 500, 750, 1,000, and 1,500 FTU/kg *S. pombe*-expressed phytase. One phytase unit (FTU) was defined as the amount of enzyme that catalyzes the release of one micromole phosphate from phytate per minute at 37°C and pH 5.5.

**Results:**

The inclusion of increasing levels of phytase in the diet linearly increased the body weight gain during days 1 to 10 (p = 0.001), 25 to 38 (p = 0.016), 39 to 45 (p = 0.018), and 1 to 45 (p = 0.004), feed intake during days 25 to 38 (p = 0.032), feed conversion ratio during days 1 to 10 (p = 0.001), 39 to 45 (p = 0.038), and 1 to 45 (p = 0.012), carcass weight (p = 0.035), toe ash (p<0.001), and apparent ileal phosphorus digestibility (p = 0.049). However, the footpad lesions score (p = 0.040) decreased linearly with the increase in phytase levels in the diet.

**Conclusion:**

Dietary supplementation of *S. pombe*-expressed phytase was beneficial to the growth performance, toe ash, apparent ileal phosphorus digestibility, and footpad lesions of broiler chicks in a dose-dependent manner.

## INTRODUCTION

Poultry receives nutrients needed for growth and production from plant-based resources, however, a big part of nutrient ingredients such as protein, phosphorus, and calcium bind with phytate [1,2], consequently reducing the availability of nutrients from the feed [1,3]. In addition, it has been reported that phytate can also impair digestive enzymes in the intestine [4,5] and downregulate the mRNA expression of ghrelin in the jejunum [6]. Therefore, degradation of phytate in feed is of great significance to save feed costs and improve animal growth performance.

Phytase has attracted wide attention because of its specific hydrolysis of phytate. It can hydrolyze phytate to release phytate bound nutrient components, thereby reducing the anti-nutritive effect of phytate [7]. It is reported that feeding broiler chicks with phytase-containing diet could improve nutrient digestibility [8,9], growth performance [10], immune status [11], and bone quality [10]. However, studies on the effects of dietary supplementation of phytase on the meat quality of broiler chicks are still limited.

Footpad condition is an important aspect of poultry welfare [12]. It causes pain in severe cases, thus impairing feed intake (FI) and growth performance [13]. It is reported that the footpad dermatitis was mainly related to the quality of bedding [14]. However, the footpad lesions also occurred in cage-reared broiler chicks [15]. Many studies have reported that supplementing phytase to the diet of floor-reared broiler chicks could improve the footpad lesions [16,17]. However, no studies have investigated the effects of phytase on the footpad lesions of cage-reared broiler chicks.

In the present study, we hypothesized that dietary supplementation of phytase could improve nutrient digestibility and toe ash, thus improving growth performance, meat quality, organ indexes, and footpad lesions score. The objective of this study was to investigate the effects of feeding broiler chicks with phytase-containing diet on growth performance, apparent ileal digestibility (AID), meat quality, organ indexes, toe ash, footpad lesions in broiler chicks.

## MATERIALS AND METHODS

This experiment was processed under the supervision of the Animal Care and Use Committee of Dankook University (Cheonan, South Korea). The relevant protocol has been approved by the above committee (DK-1-1706).

### Information of phytase

The microbial phytase (Phyzyme XP; Danisco Animal Nutrition, Marlborough, Wiltshire, UK) used in this study was in a fine granular form. It is derived from *Escherichia coli* and expressed by *Schizosaccharomyces Pombe* (*S. Pombe*; ATCC 5233). According to the European Food Safety Authority (EFSA) [18], the stability of this phytase is over 95% after storage in a 20°C environment for 6 months. The optimal pH is 4.5 [19].

One phytase unit (FTU) was defined as the amount of enzyme that catalyzes the release of one micromole phosphate from phytate per minute at 37°C and pH 5.5 [18].

### Animals and housing

A total of 390-day-old Ross 308 broiler chicks were randomly assigned to five groups based on the initial body weight (42.15 ±0.17 g). There were 6 replicate cages per treatment with 13 birds (mixed sex) per cage. The size of cage was 1.55×0.75× 0.55 m. All birds were housed in 3-floor battery cages. The temperature of room was 32°C at start and reduced by 2°C per week up to 24°C. The humidity of room was 65%. The provision of light to birds was for 24 h during days 1 to 7 and 16 h of light and 8 h of dark during days 8 to 45. There were 2 feeders and 2 nipple drinkers equipped in the cage to provide feed and water *ad libitum* to birds.

### Treatments and diets

The experimental period was 45 days, which was divided into four periods: starter, days 1 to 10; grower, days 11 to 24; finisher 1, days 25 to 38; finisher 2, days 39 to 45. Dietary treatments were based on a corn-soybean meal basal diet as control (Table 1) and basal diet supplemented with 500, 750, 1,000, and 1,500 FTU/kg *S. pombe*-expressed phytase. Phytase was mixed with 1 kg of feed by hand, and then premix was mixed with the remaining feed by using a blender to ensure homogeneity. Diets were formulated to meet the nutrient requirements recommended by Aviagen [20] and provided in mash form.

### Sample collection and measurements

#### Feed composition analysis

After homogeneous mixing, about 250 g of feed samples from each treatment diet in each period were collected in triplicate. All feed samples were dried in a 70°C oven for 72 h. Then, feed samples were ground and sieved with a 1-mm sieve. Powder feed samples were collected for feed composition analysis.

According to the procedure established by the AOAC [21], the dry matter (method 930.15), crude protein (nitrogen ×6.25; method 968.06), crude fat (method 954.02), crude ash (method 942.05), calcium (method 984.01), phosphorus (method 965.17), and crude fiber (method 991.43) composition in the diet were analyzed. Then, the representative feed samples in each group were hydrolyzed with 6 N HCl for 24 h at 110°C. An amino acid analyzer (2690 Alliance; Waters, Inc., Milford, MA, USA) was used for determining amino acid contents in the diet. Energy in feed was measured by a bomb calorimeter (Parr 6100; Parr Instrument Co., Moline, IL, USA). Phytate-P in raw materials and diets was determined using the method described by Reichwald and Hatzack [22]. Absorbance was determined using a Media spectrophotometer (Marcel Lamidey S.A., Châtillon, France) at a 519 nm wavelength. Sodium was determined in accordance with AOAC [23] using microwave plasma-atomic emission spectrometry (4100 MP-AES; Agilent Technologies, Santa Clara, USA).

#### Growth performance

All birds were weighed on days 1, 11, 25, 39, and 45 to calculate body weight gain (BWG). Cage-based FI was calculated daily. Feed conversion ratio (feed to gain ratio; FCR) was calculated based on the values of BWG and FI. Dead birds were checked daily for measuring mortality.

#### Apparent ileal digestibility

During days 38 to 45, 0.2% chromium oxide was added to the diet of birds to determine AID of crude protein, calcium, and phosphorus. At the end of the experiment, 2 birds were randomly selected from each cage and slaughtered by cervical dislocation. A portion of the small intestine from Meckel’s diverticulum proximal to the ileocecal junction as ileal samples were collected for analysis AID. Chromium concentrations were determined by atomic absorption spectrophotometry (UV-1201; Shimadzu, Kyoto, Japan). The AID was calculated relative to chromium concentrations [24].

#### Relative weight of organs

The breast muscle, liver, bursa of fabricius, abdominal fat, spleen, and gizzard from the above slaughtered birds were removed and weighed to calculate the relative weight of organs. Then, the breast muscle was stored at 2°C for measuring meat quality. The organ indexes were measured using the following equation:


$$Organ\;index=\frac{Organ\;weight}{Live\;body\;weight}\times 100\%$$

#### Meat quality

A model CR-410 Chroma meter (Konica Minolta Sensing, Inc., Osaka, Japan) was used to measure the lightness, redness, and yellowness values. The value of lightness, redness, and yellowness were calculated as the average of three positions on the surface of each sample. The pH values of each breast meat sample were measured in duplicate using a pH meter (Fisher Scientific, Pittsburgh, PA, USA). Thereafter, a 0.20 g meat sample was pressed at 20.7 MPa for 3 minutes on a 125-mm-diameter piece of filter paper. The areas of the pressed sample and the expressed moisture were delineated and then determined using a digitizing area-line sensor (MT-10S; M.T. Precision Co. Ltd., Tokyo, Japan) to calculate the water holding capacity (WHC). About 5 g meat sample was stored in plastic bags and bath in 100°C water for 5 minutes to measure cooking loss. Then samples were cooled at room temperature. Cooking loss was calculated as:


$$\text{Cooking loss }(\%)=\frac{sample\;weight\;before\;cooking-sample\;weight\;after\;cooking}{sample\;weight\;before\;cooking})\times 100\%$$

About 2 g of meat sample was suspended in a zipper bag in a 4°C environment and weighed on days 1, 3, 5, and 7 to calculate the drip loss.

#### Toe ash

The left and right middle toes were excised from the above slaughtered birds and pooled separately to yield four samples of toes per replicate cage. These were averaged for the statistical analysis of the toe ash data. The composite samples were dried overnight at 100°C, extracted in ether for 6 h, and ashed in a muffle furnace for 18 h at 600°C [25].

#### Footpad lesions score

Lesions score of footpad dermatitis was measured on day 44 for all birds. Footpad dermatitis was scored on a 4-point scale: score 0, no lesions on the footpad; score 1, small lesions of the footpad epithelium (<1 cm); score 2, larger lesions (>1 cm); and score 3, dorsal swelling visible (bumble foot) [10].

### Statistical analysis

All data were statistically analyzed using the General Linear Model procedure (SAS Inst. Inc., Cary, NC, USA) in a randomized completely block design. The replicate cage was used as the experimental unit. Orthogonal contrasts were used to examine the linear and quadratic effects in response to increasing the dietary supplementation of phytase. Variability in the data was expressed as the standard error of means, p<0.05 was considered statistically significant.

## RESULTS

The BWG during days 1 to 10 (p = 0.001), 25 to 38 (p = 0.016), 39 to 45 (p = 0.018), and 1 to 45 (p = 0.004), FI during days 25 to 38 (p = 0.032), and FCR during days 1 to 10 (p = 0.001), 39 to 45 (p = 0.038), and 1 to 45 (p = 0.012) increased linearly as the levels of phytase increased in the diet. However, phytase supplementation did not affect the mortality (Table 2).

Feeding broiler chicks with phytase-containing diet linearly increased the AID of phosphorus (p = 0.049), while did not affect the AID of calcium and crude protein (Table 3).

The carcass weight linearly increased with the increase in phytase levels in the diet (p = 0.035). However, dietary supplementation of phytase had no effects on the relative weight of breast muscle, liver, bursa of fabricius, abdominal fat, spleen, and gizzard (Table 4).

The meat quality including meat color (lightness, redness, and yellowness), pH, drip loss, cooking loss, and WHC (Table 5) was not affected in broiler chicks fed the diet supplemented with phytase.

The footpad lesions score decreased linearly with the graded levels of phytase supplemented in the diet (p = 0.040). In addition, the toe ash increased linearly as the levels of phytase increased in the diet (p<0.001) (Table 6).

## DISCUSSION

The hydrolysis of phytate by phytase in the upper digestive tract is important for releasing phytate-bound nutrient ingredients [26,27]. The pH of the forestomach in poultry is 4 to 5 [28]. The suitable pH of *S. pombe*-expressed phytase used in the present study was 4.5. The common parameters to evaluate the efficacy of phytase include phosphorus digestibility, growth performance, and bone ash [4]. The improvement of AID of phosphorus, growth performance, and toe ash by *S. pombe*-expressed phytase supplementation was observed in this study. Therefore, we considered that the *S. pombe*-expressed phytase was effective *in vivo*.

Earlier studies have proved that feeding broiler chicks with phytase-containing diet could improve their growth performance [29,30] which agrees with the findings of the present study. The mechanism of phytase supplementation improving growth performance of broiler chicks was related to the event of phytate hydrolysis by phytase, which releases the phytate-bound nutrient ingredients [31,32], thus improving nutrient digestibility and resulting in a decrease of FCR and an increase of BWG in birds [5,33,34].

It is reported that supplementing phytase in diet could improve the nutrient digestibility in broiler chicks [35,36]. In this study, feeding broiler chicks with phytase-containing diet led to an increase of AID of phosphorus, whereas did not affect the AID of crude protein and calcium. The efficacy of phytase in releasing nutrient ingredients from phytate depends on the binding degree between nutrient ingredients and phytate [37,38]. In plant-based feed, more than 50% of phytate was bound with phosphorus [39]. Therefore, the hydrolysis of phytate by phytase resulted in a higher release of phosphorus than protein and calcium [40]. In this study, we considered that the supplementation of *S. pombe*-expressed phytase could lead to the increase of phytate hydrolysis, which was manifested in the increase of AID of phosphorus. The lack of effects of phytase on calcium and crude protein digestibility could be explained by the difference of phytate binding degree between phosphorus with calcium and protein [40]. Similarly, many studies have reported that feeding broiler chicks with 250 to 1,000 FTU/kg *S. pombe*-expressed phytase had positive effects on the AID of phosphorus but did not affect the AID of calcium and crude protein [34,41–43]. In brief, dietary supplementation of *S. pombe*-expressed phytase could degrade phytate, manifested in the increase of the AID of phosphorus.

In addition, the toe ash is one of the sensitive indicators of phosphorus utilization [44,45]. It is reported that dietary supplementation of *S. pombe*-expressed phytase could increase the toe ash [25,44,46,47]. In the present study, the improvement of toe ash was also observed in birds receiving diets with increasing levels of phytase supplementation. Therefore, we considered that the addition of *S. pombe*-expressed phytase in the diet could increase phosphorus utilization, manifested in the improvement of toe ash.

Therefore, in the present study, dietary supplementation of *S. pombe*-expressed phytase improvement of the growth performance of broiler chicks was related to the improvement of phosphorus utilization, manifested in the increase of the AID of phosphorus and the toe ash, which was consistent with the studies of Taheri and Mirisakhani [48] and Hajimohammadi et al [34]. On the other hand, Liu et al [6] reported that phytase supplementation could downregulate the somatostatin gene and upregulate the ghrelin gene, which may be the molecular mechanism whereby phytase improves growth performance. In general, dietary supplementation of *S. pombe*-expressed phytase had positive effects on the growth performance of broiler chicks.

The improvement of carcass weight was observed in feeding broiler chicks with *S. pombe*-expressed phytase-containing diet. This can be predicted because the increase of carcass weight was related to the increase of body weight, a higher body weight corresponds to a higher carcass weight [48]. Similarly, several studies have reported that feeding broiler chicks with phytase-containing diet improved body weight, thus leading to a higher carcass weight [34,48,49]. However, dietary supplementation of phytase did not affect the relative weight of breast weight, liver, bursa of fabricius, abdominal fat, spleen, and gizzard. Some studies also reported that feeding broiler chicks with phytase-containing diet did not affect the relative weight of breast muscle [29,48], liver [30,50], bursa of fabricius [34,51], abdominal fat [49,50], spleen [33,52], and gizzard [30,53]. Therefore, we considered that the development of organs was not sensitive to the supplementation of graded levels of *S. pombe*-expressed phytase.

The major indicators in the meat quality of breast muscle of broiler chicks include meat color, WHC, drip loss, and cooking loss, which are important meat attributes for the consumers purchasing the product as well as to the processors of value-added meat products [54]. The pH value is a direct reflection of muscle acid content, which affects cooking loss [55], meat color [56], WHC [55], and drip loss [57]. Studies on the effects of dietary supplementation of *S. pombe*-expressed phytase on meat quality are still limited. Attia et al [36] reported that dietary supplementation of *S. pombe*-expressed phytase did not affect the color of meat. In this study, dietary supplementation of *S. pombe*-expressed phytase also did not affect the meat quality, which probably related to the fact that the pH of meat was not affected by phytase supplementation. Therefore, dietary supplementation of *S. pombe*-expressed phytase was not beneficial to improve the meat quality, but also did not induce any negative effects.

In the present study, dietary supplementation of *S. pombe*-expressed phytase could ameliorate the footpad lesions. The effects of phytase supplementation on the amelioration of footpad lesions have been widely reported [16,17,58]. However, in the above studies, broiler chicks were floor-reared. They reported that the mechanism of ameliorating footpad lesions through supplementing phytase was related to the prevention of wet litter [59]. However, in the present study, broiler chicks were reared in the battery-cage. This means birds were provided with litter. Therefore, it is notable that litter quality improvement is not the only factor that improves footpad lesions and other mechanisms influenced by phytase supplementation must have played a role in reducing footpad lesions. Delezie et al [16] reported that feeding floor-reared broiler chicks with *Pichia pastoris*-expressed phytase-containing diet did not affect the litter quality but significantly ameliorated the footpad lesions. In humans, Mukovozov et al [60] reported that atopic dermatitis was related to poor bone health. We speculated that the improvement of bone quality and phosphorus digestibility may be beneficial to the amelioration of footpad lesions. Studies reported that footpad lesions improved by phytase supplementation also corresponds to the improvement of bone quality and phosphorus digestibility [16,58,59]. Therefore, the improvement of footpad lesions by *S. pombe*-expressed phytase supplementation may be associated with the improvement of phosphorus digestibility and toe ash. However, more experiments are needed to be further investigated. In brief, we considered that dietary supplementation of *S. pombe*-expressed phytase could reduce footpad lesions, which is probably related to the improvement of phosphorus digestibility and toe ash.

## CONCLUSION

In this study, we found that dietary supplementation of graded levels of *S. pombe*-expressed phytase could improve growth performance, AID of phosphorus, toe ash, footpad lesions in a dose-dependent manner. Therefore, *S. pombe*-expressed phytase supplementation has great significance for improving the growth performance and footpad lesions of broiler chicks, which was related to the increase of phosphorus utilization, manifested in the improvement of phosphorus digestibility and toe ash.

## Figures and Tables

**Table 1:** Composition and nutrient levels of the experimental basal diet, (%, as-fed basis)

Items	Feeding phases			

	Starter (d 0 – 10)	Grower (d 11 – 24)	Finisher 1 (d 24 – 38)	Finisher 2 (d 38 – 45)
Ingredients (%)				
Corn	53.87	55.92	59.73	60.41
Soybean meal, 48%	33.12	26.10	22.18	21.25
Canola meal	5.00	5.00	5.00	5.00
Brown rice	5.00	10.00	10.00	10.00
Yellow grease	-	0.12	0.50	1.04
Tricalcium phosphate	0.83	0.68	0.39	0.23
Limestone	0.86	0.91	0.95	0.87
Vitamin and trace mineral premix^1)^	0.35	0.35	0.35	0.35
DL-Methionine, 99%	0.29	0.29	0.27	0.21
Salt	0.24	0.24	0.25	0.28
L-Lysine HCl	0.23	0.18	0.18	0.17
L-Threonine, 98.5%	0.08	0.08	0.07	0.06
Choline-Cl, 60%	0.08	0.08	0.08	0.08
Sodium bicarbonate	0.05	0.05	0.05	0.05
Total	100.00	100.00	100.00	100.00
Calculated value (%)				
Arginine	1.48	1.28	1.15	1.12
Lysine	1.38	1.16	1.06	1.03
Methionine	0.62	0.59	0.54	0.49
Methionine+cysteine	1.00	0.94	0.86	0.81
Leucine	1.88	1.67	1.56	1.53
Isoleucine	0.99	0.85	0.77	0.75
Threonine	0.91	0.81	0.75	0.73
Valine	1.08	0.95	0.87	0.85
Available phosphorus	0.45	0.44	0.38	0.35
Analyzed composition (%)				
Metabolizable energy (MJ/kg)	12.63	12.92	13.20	13.39
Crude protein	23.10	20.21	18.62	18.19
Crude fat	3.01	3.24	3.71	4.26
Crude fiber	3.20	3.13	3.08	3.06
Dry matter	85.08	85.06	85.28	85.58
Crude ash	4.89	4.58	4.20	3.95
Calcium	0.90	0.85	0.75	0.66
Total phosphorus	0.81	0.81	0.74	0.71
Sodium	0.21	0.21	0.21	0.21
Phytate phosphorus	0.27	0.27	0.26	0.26
Digestible lysine	1.25	1.05	0.96	0.93
Digestible methionine	0.59	0.57	0.52	0.47
Digestible cysteine	0.32	0.29	0.28	0.28
Digestible sulfur amino acid	0.92	0.87	0.80	0.75
Digestible threonine	0.78	0.69	0.64	0.62
Digestible tryptophan	0.22	0.19	0.17	0.17
Digestible valine	0.95	0.81	0.74	0.72
Digestible leucine	1.71	1.50	1.40	1.37
Digestible isoleucine	0.88	0.74	0.66	0.65
Digestible arginine	1.36	1.13	1.02	1.00

1) Provided per kg of complete diet: 37.5 mg Zn (as ZnSO_4_); 37.5 mg Mn (as MnO_2_); 37.5 mg Fe (as FeSO_4_·7H_2_O); 3.75 mg Cu (as CuSO_4_·5H_2_O); 0.83 mg I (as KI); and 0.23 mg Se (as Na_2_SeO_3_·5H_2_O), 15,000 IU of vitamin A, 3,750 IU of vitamin D_3_, 37.5 IU of vitamin E, 2.55 mg of vitamin K_3_, 3 mg of thiamin, 7.5 mg of riboflavin, 4.5 mg of vitamin B_6_, 24 ug of vitamin B_12_, 51 mg of niacin, 1.5 mg of folic acid, 0.2 mg of biotin, and 13.5 mg of Ca-pantothenate.

**Table 2:** Effects of feeding broiler chicks with *Schizosaccharomyces pombe*-expressed phytase-containing diet on growth performance

Items	*S. pombe* phytase (FTU/kg)					SEM	p-value	
	
	0	500	750	1,000	1,500		Linear	Quadratic
IBW (g)	41.88	42.21	42.23	42.21	42.23	0.166	0.195	0.293
BWG (g)								
Days 1–10	232.29^c^	238.20^bc^	247.31^ab^	246.75^ab^	251.14^a^	3.863	0.001	0.390
Days 11–24	613.20	602.24	610.59	624.09	623.99	10.852	0.220	0.516
Days 25–38	1,092.88^b^	1,097.61^b^	1,112.07^ab^	1,127.33^ab^	1,143.10^a^	19.578	0.016	0.705
Days 39–45	613.79^b^	619.96^ab^	626.95^ab^	631.86^ab^	646.71^a^	11.919	0.018	0.678
Days 1–45	2,552.17^b^	2,558.01^b^	2,596.91^ab^	2,630.04^ab^	2,664.94^a^	28.637	0.004	0.631
FI (g)								
Days 1–10	368.77	370.92	371.69	375.69	369.54	5.441	0.718	0.518
Days 11–24	1,151.33	1,119.19	1,148.05	1,143.96	1,141.59	15.430	0.915	0.650
Days 25–38	2,332.17^b^	2,339.71^ab^	2,352.46^ab^	2,359.20^a^	2,359.35^a^	10.113	0.032	0.589
Days 39–45	1,154.06	1,170.51	1,171.62	1,181.52	1,166.67	10.567	0.291	0.189
Days 1–45	5,006.32	5,000.33	5,043.82	5,060.36	5,037.14	26.146	0.157	0.537
FCR								
Days 1–10	1.59^a^	1.56^ab^	1.50^bc^	1.52^bc^	1.47^c^	0.021	0.001	0.648
Days 11–24	1.88	1.86	1.88	1.83	1.83	0.037	0.312	0.800
Days 25–38	2.13	2.14	2.12	2.10	2.07	0.039	0.090	0.548
Days 39–45	1.89^a^	1.89^a^	1.87^ab^	1.87^ab^	1.80^b^	0.033	0.038	0.275
Days 1–45	1.96^a^	1.96^a^	1.94^ab^	1.93^ab^	1.89^b^	0.020	0.012	0.430
Mortality (%)	0.14	0.11	0.14	0.12	0.11	0.032	0.652	0.899

SEM, standard error of the mean; IBW, initial body weight; BWG, body weight gain; FI, feed intake; FCR, feed conversion ratio.

a–c Different superscripts within a row indicate a significant difference (p<0.05).

**Table 3:** Effects of feeding broiler chicks with *Schizosaccharomyces pombe*-expressed phytase-containing diet on apparent ileal digestibility

Items (%)	*S. pombe* phytase (FTU/kg)					SEM	p-value	
	
	0	500	750	1,000	1,500		Linear	Quadratic
Calcium	57.20	58.89	56.31	56.39	55.84	1.597	0.309	0.765
Phosphorus	45.95^b^	47.21^ab^	46.34^ab^	46.71^ab^	48.45^a^	2.292	0.049	0.410
Crude protein	65.42	66.66	66.37	67.20	67.94	1.792	0.400	0.757

SEM, standard error of the mean.

a,b Different superscripts within a row indicate a significant difference (p<0.05).

**Table 4:** Effects of feeding broiler chicks with *Schizosaccharomyces pombe*-expressed phytase-containing diet on organ indexes

Items	*S. pombe* phytase (FTU/kg)					SEM	p-value	
	
	0	500	750	1,000	1,500		Linear	Quadratic
Carcass weight (g)	2,441.50^b^	2,529.50^ab^	2,548.50^ab^	2,503.13^ab^	2,606.00^a^	49.586	0.035	0.834
Breast muscle (%)	19.10	19.10	19.26	19.43	19.42	0.518	0.555	0.995
Liver (%)	2.91	2.97	3.01	2.97	2.93	0.118	0.926	0.548
Bursa of fabricius (%)	0.15	0.15	0.14	0.14	0.15	0.010	0.673	0.577
Abdominal fat (%)	3.22	3.12	3.17	3.12	3.11	0.181	0.717	0.897
Spleen (%)	0.11	0.10	0.09	0.11	0.11	0.009	0.589	0.246
Gizzard (%)	1.33	1.35	1.33	1.36	1.34	0.062	0.876	0.954

SEM, standard error of the mean.

a,b Different superscripts within a row indicate a significant difference (p<0.05).

**Table 5:** Effects of feeding broiler chicks with *Schizosaccharomyces pombe*-expressed phytase-containing diet on meat quality

Items	*S. pombe* phytase (FTU/kg)					SEM	p-value	
	
	0	500	750	1,000	1,500		Linear	Quadratic
Meat color								
Lightness	58.99	58.17	57.06	57.79	57.66	1.077	0.376	0.429
Redness	11.59	12.06	12.05	11.44	12.22	0.554	0.717	0.997
Yellowness	14.96	15.30	15.53	15.46	15.32	1.046	0.793	0.750
pH	5.56	5.41	5.40	5.43	5.56	0.089	0.466	0.260
Drip loss (%)								
Day 1	3.02	3.39	3.21	3.07	3.29	0.225	0.758	0.758
Day 3	6.34	6.21	6.25	6.29	6.23	0.263	0.859	0.891
Day 5	11.48	11.25	11.31	11.12	11.13	0.374	0.482	0.877
Day 7	12.20	12.40	12.28	11.99	12.19	0.663	0.841	0.946
Cooking loss (%)	18.58	18.46	18.55	18.40	18.54	0.605	0.941	0.900
WHC (%)	57.43	57.13	57.46	57.34	57.28	0.866	0.973	0.992

SEM, standard error of the mean; WHC, water-holding capacity.

**Table 6:** Effects of feeding broiler chicks with *Schizosaccharomyces pombe*-expressed phytase-containing diet on toe ash and footpad lesions score

Items	S. pombe phytase (FTU/kg)					SEM	p-value	
	
	0	500	750	1,000	1,500		Linear	Quadratic
Footpad lesions score	2.50^a^	2.50^a^	2.25^ab^	2.25^ab^	1.88^b^	0.253	0.040	0.552
Toe ash (%)	11.91^b^	12.39^b^	12.52^b^	14.20^a^	15.06^a^	0.459	<0.001	0.192

SEM, standard error of the mean.

a,b Different superscripts within a row indicate a significant difference (p<0.05).
